# Effect of Body Mass Index on Perioperative Outcomes in Minimally Invasive Oblique Lateral Lumbar Interbody Fusion versus Open Fusions: A Multivariant Analysis

**DOI:** 10.7759/cureus.2288

**Published:** 2018-03-08

**Authors:** Hamid Abbasi, Alex Grant

**Affiliations:** 1 Tristate Brain and Spine Institute; 2 Fairview Health Services, Fairview Ridges Hospital

**Keywords:** operative surgical procedures, minimally invasive, spinal fusion, lumbar spine, disk disease, transforaminal lumbar interbody fusion, fusion, obesity

## Abstract

Background

Obesity is an increasing public health concern associated with increased perioperative complications and expense in lumbar spine fusions. While open and mini-open fusions such as transforaminal lumbar interbody fusion (TLIF) and minimally invasive TLIF (MIS-TLIF) are more challenging in obese patients, new MIS procedures like oblique lateral lumbar interbody fusion (OLLIF) may improve perioperative outcomes in obese patients relative to TLIF and MIS-TLIF.

Purpose

The purpose of this study is to determine the effects of obesity on perioperative outcomes in OLLIF, MIS-TLIF, and TLIF.

Study design

This is a retrospective cohort study.

Patient sample

We included patients who underwent OLLIF, MIS-TLIF, or TLIF on three or fewer spinal levels at a single Minnesota hospital after conservative therapy had failed. Indications included in this study were degenerative disc disease, spondylolisthesis, spondylosis, herniation, stenosis, and scoliosis.

Outcome measures

We measured demographic information, body mass index (BMI), surgery time, blood loss, and hospital stay.

Methods

We performed summary statistics to compare perioperative outcomes in MIS-TLIF, OLLIF, and TLIF. We performed multivariate regression to determine the effects of BMI on perioperative outcomes controlling for demographics and number of levels on which surgeries were operated.

Results

OLLIF significantly reduces surgery time, blood loss, and hospital stay compared to MIS-TLIF, and TLIF for all levels. MIS-TLIF and TLIF do not differ significantly except for a slight reduction in hospital stay for two-level procedures. On multivariate analysis, a one-point increase in BMI increased surgery time by 0.56 ± 0.47 minutes (p = 0.24) in the OLLIF group, by 2.8 ± 1.43 minutes (p = 0.06) in the MIS-TLIF group, and by 1.7 ± 0.43 minutes (p < 0.001) in the TLIF group. BMI has positive effects on blood loss for TLIF (p < 0.001) but not for OLLIF (p = 0.68) or MIS-TLIF (p = 0.67). BMI does not have significant effects on length of hospital stay for any procedure.

Conclusions

Obesity is associated with increased surgery time and blood loss in TLIF and with increased surgery time in MIS-TLIF. Increased surgery time may be associated with increased perioperative complications and cost. In OLLIF, BMI does not affect perioperative outcomes. Therefore, OLLIF may reduce the disparity in outcomes and cost between obese and non-obese patients.

## Introduction

Obesity is a major public health concern around the world. According to the World Health Organization and the National Institute of Health, individuals with a body mass index (BMI) of 30.0 or above are considered obese, and those with a BMI of 40.0 are considered morbidly obese. Today, 37.7% of Americans are obese, and 7.7% are morbidly obese [1]. Obesity is associated with increased all-cause mortality [2] and increased risk for a wide variety of health conditions including lower back pain [3]. Lower back pain remains one of the more prevalent and expensive health conditions in the Western world [4,5] with up to 80% of all people suffering from it at some point in life. According to a recent meta-analysis, obesity is associated with an increased prevalence of lower back pain (odds ratio [OR] = 1.33), and an even larger increase in the likelihood of seeking care for lower back pain (OR = 1.53) [6]. Lower back pain may increase sedentary lifestyle, which can lead to further increases in BMI.

Lumbar fusions are one of the mainstay surgical treatments for lower back pain and have become one of the more common surgical procedures in the United States [7]. Fusions performed on obese patients are associated with increased complication rates mostly related to wound site infections [8,9]. As a result, obese fusion patients require longer hospital stays and are more expensive to treat than non-obese patients [9]. Spine surgery in obese patients is generally considered more technically challenging than in non-obese patients. Some surgeons may refrain from offering surgical treatment to patients with higher BMI due to concerns about these technical challenges.

In this study, we compare the impact of obesity on perioperative outcomes in patients who underwent transforaminal lumbar interbody fusion (TLIF), minimally invasive-TLIF (MIS-TLIF), and oblique lateral lumbar interbody fusion (OLLIF). TLIF is the most commonly performed open fusion technique on the lumbar spine today. In 2005, MI-TLIF was described as a mini-open approach to TLIF [10]. OLLIF is a recent innovation for performing lumbar fusions [11]. In OLLIF, the disc is approached through Kambin’s triangle using bilateral fluoroscopic imaging and electrophysiology to guide the approach. Variations of OLLIF enable a surgeon to approach technically difficult areas such as L5-S1 [12] and the thoracic region [13].

MIS procedures like OLLIF and MIS-TLIF reduce the surgical morbidity associated with open spinal fusions because MIS approaches decrease the disruption of adjacent muscle and connective tissue [14]. MIS lumbar fusions also decrease blood loss and infection rates relative to open procedures, and some MIS fusions, including OLLIF, substantially reduce surgery time [11,15]. However, mini-open procedures like MI-TLIF increase surgery time relative to open TLIF because the same procedure is performed through a smaller incision [16], making MI-TLIF particularly challenging in obese patients.

MIS fusions of the lumbar spine have been shown to be effective in obese patients [15,17,18], but scant evidence is available on how obesity impacts the benefits of MIS fusions relative to open fusions. Here, we compare the effects of obesity on perioperative outcomes for TLIF, MIS-TLIF, and OLLIF in a series of 321 patients in a single hospital setting.

## Materials and methods

Study design

This study is a retrospective case review of 68 OLLIF patients, 225 TLIF patients, and 28 MIS-TLIF patients. All surgeries were performed at the same Minnesota hospital by six surgeons. The cases were performed between January 2015 and September 2017. This study was determined to be exempt from institutional review board (IRB) approval by Pearl IRB on 12/29/2017.

Patient selection

Patients were considered candidates for surgery if conservative therapy had failed. Indications included in this study were degenerative disc disease, spondylolisthesis, spondylosis, herniation, stenosis, and scoliosis. Patients were included in this study if their surgery involved three or fewer levels. Patient demographics are provided in Table 1.

**Table 1:** Patient characteristics. MIS-TLIF: Minimally invasive transforaminal lumbar interbody fusion; OLLIF: Oblique lateral lumbar interbody fusion; SD: Standard deviation; TLIF: Transforaminal lumbar interbody fusion.

	OLLIF	MIS-TLIF	TLIF	p
N	68	28	225	
# of Levels (%)				0.002
1	26 (38.2)	20 (71.4)	124 (55.1)	
2	24 (35.3)	8 (28.6)	73 (32.4)	
3	18 (26.5)	0 (0.0)	28 (12.4)	
BMI (mean (SD))	30.81 (6.17)	28.75 (6.34)	30.56 (5.84)	0.272
Obesity (%)				0.350
Morbid	6 (8.8)	2 (7.1)	15 (6.7)	
Obese	31 (45.6)	7 (25.0)	92 (40.9)	
Overweight	18 (26.5)	12 (42.9)	85 (37.8)	
Normal	13 (19.1)	7 (25.0)	33 (14.7)	
Male (%)	35 (51.5)	10 (35.7)	104 (46.2)	0.369
Age (mean (SD))	54.66 (16.34)	58.21 (8.99)	59.64 (13.00)	0.029

Outcome measures

Anesthesia times, blood loss, and fluoroscopy times were recorded by clinic staff and entered into the electronic medical records (EMR) database immediately after surgery.

Statistical analysis

Data were collected in the hospital EMR and exported for analysis. The differences between study groups were tested using analysis of variance (ANOVA) with equal variance assumptions for continuous variables and Chi-Squared tests with Yates correction for continuity. To determine the effects of BMI on surgery time, multivariate regression was performed. Statistical analysis was performed in R Version 3.4.1.

## Results

Study groups did not differ significantly in terms of gender or BMI (Table 1). OLLIF patients were slightly younger than MIS-TLIF and TLIF patients. The distribution of surgical levels differed significantly between the three procedures. To account for these differences between the study groups, we corrected for number of levels, age, and sex in the subsequent multivariate analysis.

Summary statistics for the two study groups are displayed in Table 2. OLLIF is significantly faster than TLIF and MIS-TLIF for all levels and all patients. For a single-level procedure, OLLIF decreases the average surgery time by 64% and 61% relative to TLIF and MIS-TLIF, respectively (pairwise T-Tests p < 0.001). Similarly, single-level OLLIF reduces average blood loss by 60% and 48% and average hospital stay by 52% and 41% relative to TLIF and MIS-TLIF, respectively (pairwise T-Tests p < 0.001). The perioperative outcomes of TLIF and MIS-TLIF do not differ significantly, except for a slight reduction in hospital stay for two-level MIS-TLIF.

**Table 2:** Perioperative outcomes grouped by number of levels. All values are mean (SD). *Difference significant relative to OLLIF (p < 0.05). ^†^Difference significant relative to MIS-TLIF (p < 0.05). MIS-TLIF: Minimally invasive transforaminal lumbar interbody fusion; OLLIF: Oblique lateral lumbar interbody fusion; SD: Standard deviation; TLIF: Transforaminal lumbar interbody fusion.

	OLLIF	MIS-TLIF	TLIF	p
1 Level				
N	26	20	124	
Surgery time (min)	41.58^†^ (12.88)	105.37* (47.24)	115.71* (34.90)	<0.001
Blood loss (mL)	52.15 (45.46)	101.25 (125.78)	131.49* (124.87)	0.007
Hospital stay (days)	1.35^†^ (0.63)	2.30* (1.26)	2.81* (1.02)	<0.001
2 Levels				
N	24	8	73	
Surgery time (min)	76.38^†^ (32.41)	129.62* (29.68)	155.03* (43.83)	<0.001
Blood loss (mL)	118.75 (115.42)	268.75 (232.90)	234.11* (184.06)	0.015
Hospital stay (days)	1.92 (0.78)	2.25 (0.71)	3.05*^†^ (0.80)	<0.001
3 Levels				
N	18	0	28	
Surgery time (min)	94.06 (18.45)		195.64* (41.61)	<0.001
Blood loss (mL)	164.06 (124.02)		407.14* (219.83)	<0.001
Hospital stay (days)	3.06 (1.16)		3.39 (1.13)	0.335

**Figure 1:**
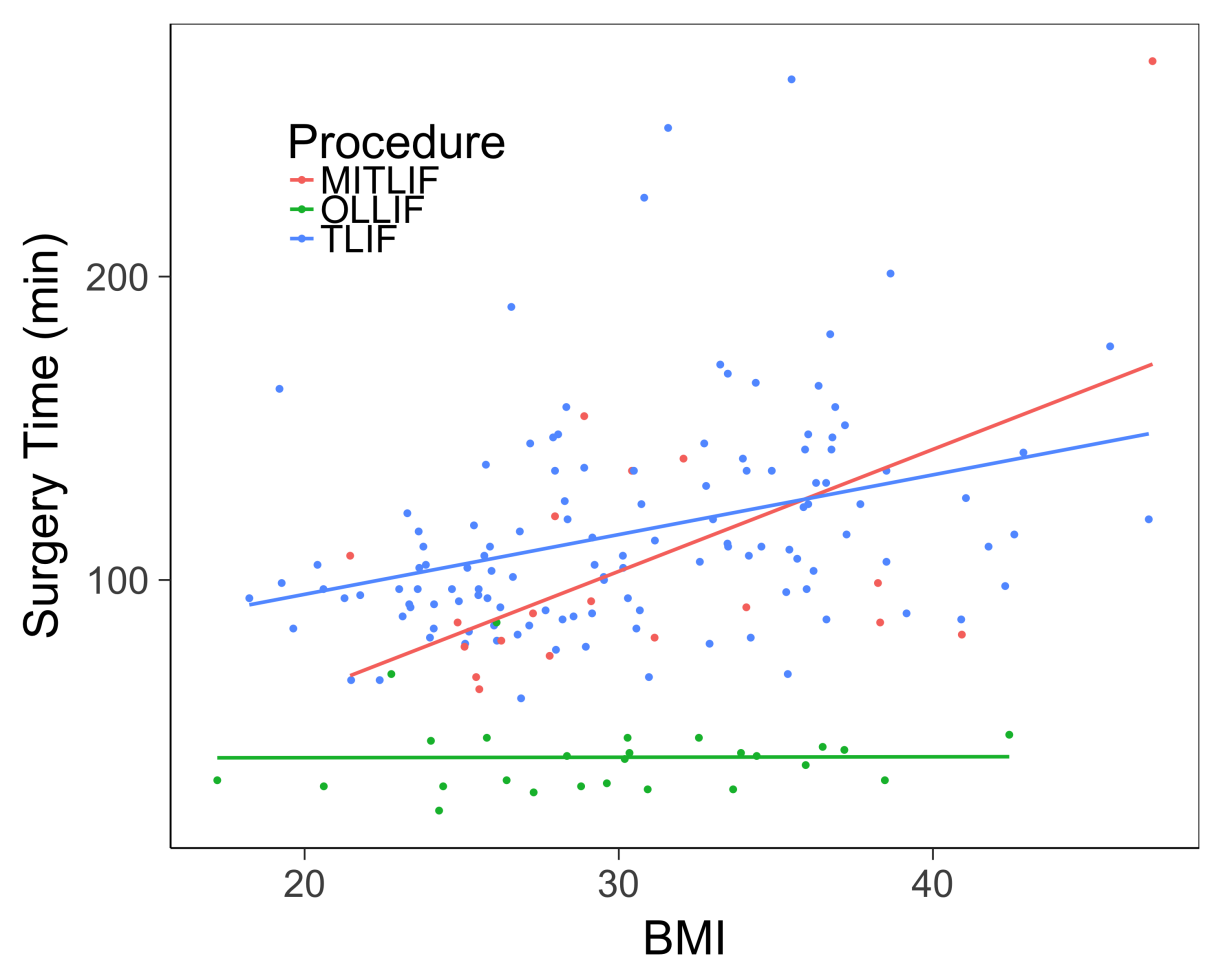
BMI vs. surgery time for single level procedures. The surgery time and BMI of each 1 level procedure for OLLIF (green), MI-TLIF (red), and TLIF (blue). Univariate regression lines are plotted for reference. BMI: Body mass index; MIS-TLIF: Minimally invasive transforaminal lumbar interbody fusion; OLLIF: Oblique lateral lumbar interbody fusion; TLIF: Transforaminal lumbar interbody fusion.

We ran a multivariate regression to determine the effects of BMI on surgery time, blood loss, and length of stay, controlling for age, sex, and the number of levels fused. The effect of BMI on surgery time is shown in Table 3 and the relationship between surgery time and BMI for single-level fusions is illustrated in Figure 1. BMI has positive effects on surgery time for all procedures, but this effect varies in size and statistical significance. More specifically, a one-point increase in BMI increased surgery time by 0.56 ± 0.47 minutes (p = 0.24) in the OLLIF group, by 2.8 ± 1.43 minutes (p = 0.06) in the MIS-TLIF group, and by 1.7 ± 0.43 minutes (p < 0.001) in the TLIF group. Table 4 demonstrates the effect of BMI on blood loss. BMI has positive effects on blood loss for TLIF (p < 0.001) but not for OLLIF (p = 0.68) or MIS-TLIF (p = 0.67). BMI does not have significant effects on length of hospital stay for any procedure (results not shown).

**Table 3:** Regression coefficients for the effect of BMI on surgery time. ^†^p < 0.1 *p < 0.05 **p < 0.001 BMI: Body mass index; MIS-TLIF: Minimally invasive transforaminal lumbar interbody fusion; OLLIF: Oblique lateral lumbar interbody fusion; SD: Standard deviation; TLIF: Transforaminal lumbar interbody fusion.

Dependent Variable: Surgery Time (min)	OLLIF	MIS-TLIF	TLIF
BMI	0.556	2.827^†^	1.711**
	(0.467)	(1.426)	(0.427)
F-Statistic	14.708** (df = 4; 63)	1.680 (df = 4; 22)	37.780** (df = 4; 220)
R2	0.483	0.234	0.407

 

**Table 4:** Regression coefficients for the effect of BMI on blood loss. *p < 0.05 **p < 0.001 BMI: Body mass index; MIS-TLIF: Minimally invasive transforaminal lumbar interbody fusion; OLLIF: Oblique lateral lumbar interbody fusion; SD: Standard deviation; TLIF: Transforaminal lumbar interbody fusion.

Dependent Variable: Blood Loss (mL)	OLLIF	MIS-TLIF	TLIF
BMI	-0.827	-2.21	5.389**
	-1.977	-5.073	-1.813
F-Statistic	3.619* (df = 4; 23)	3.759** (df = 4; 63)	21.016** (df = 4; 220)
R2	0.386	0.193	0.276

## Discussion

We found that OLLIF significantly reduces blood loss relative to TLIF, a finding that is consistent with the literature on minimally invasive fusions [14]. MIS fusions decrease blood loss due to the use of smaller incisions, reduced dissections of muscles and soft tissue, and avoidance of epidural bleeding. Additionally, OLLIF does not require osteotomies during approach, further limiting blood loss. Unlike other MIS fusions, OLLIF also reduces surgery time relative to open equivalents. This finding is consistent with the results from our previous study [11]. This result is important because a clear correlation has been established between surgery time and surgery/anesthesia-related complications [19-21]. Therefore, OLLIF may avoid the increase in complication rates generally associated with surgery in obese patients.

In contrast to OLLIF, MIS-TLIF does not significantly reduce surgery time relative to TLIF. This finding is consistent with the literature on mini-open approaches as MIS-TLIF is not generally associated with reduced surgery time [16]. We also found no statistically significant difference in blood loss between MIS-TLIF and TLIF. This finding is not consistent with the literature on mini-open fusions. However, this result may be due to the small size of the MIS-TLIF study group. Notably, there were only eight cases in the two-level MIS-TLIF group, with one patient losing 800 mL of blood, which skewed the overall statistics.

For TLIF and MIS-TLIF, surgery times increase significantly with increasing BMI, which is consistent with other studies [22]. For OLLIF however, the effect of BMI on surgery time was much smaller and not statistically significant. In TLIF, the spine must be exposed and directly visualized. For obese patients, more time must be spent dissecting tissue during approach, and closure may also require more time. For OLLIF on the other hand, no dissection of soft tissues is required. The probe that is used to access the disc space can be advanced quickly through the subcutaneous layers of fat, muscle, and fascia. The surgeon then inserts the working tube, creating a sealed connection from the skin to the disk space. As subsequent tools are fed through the working tube, obesity does not affect the difficulty of the OLLIF procedure. The OLLIF approach may even be easier when performed on an obese patient because the perineural fat creates a cushion around the nerve root, lowering the risk of nerve injury in moderately obese patients.

Other MIS fusions approach the spine anteriorly. Anterior approaches have been demonstrated to improve patient outcomes relative to posterior approaches because posterior muscle groups are left intact [23]. Unfortunately, access to the lumbar spine from an anterior angle is difficult because the surgeon must traverse abdominal or retroperitoneal structures, which often requires an access surgeon. In morbidly obese patients, these approaches may be extremely difficult. OLLIF combines the advantages of anterior fusions with a simpler approach that is not significantly affected by obesity.

Previous studies have shown that the perioperative costs of spinal fusions are increased for obese patients [9], while minimally invasive procedures may reduce costs due to decreased surgery time and hospital stay [24].

Limitations

This study’s key limitation is that no clinical outcome data were collected. Therefore, it remains to be seen whether reduced surgery time would also deliver improved patient outcomes. Furthermore, the study’s setting was a single hospital in Minnesota. Further evaluation is required to understand whether the results of this study can be generalized and applied to other settings. Finally, this study is also limited because it is a retrospective analysis.

## Conclusions

Perioperative outcomes in OLLIF are not affected by obesity. Therefore, OLLIF may significantly reduce the additional costs associated with performing lumbar fusions on obese patients.
